# EBV-DNA viral load and lymphocyte subsets correlation in pediatric infectious mononucleosis with hepatic injury a retrospective cohort study

**DOI:** 10.3389/fped.2026.1735179

**Published:** 2026-03-25

**Authors:** Zhi Yang, Zong-Tao Ma, Qing-Feng Fang, Bi-Quan Chen, Yuan Xu

**Affiliations:** 1 Department of Pediatric Infectious Disease, Anhui Provincial Children’s Hospital, Hefei, China; 2 Department of Pediatric, Lixin Hospital of Chinese Medicine, Bozhou, Anhui, China; 3 Department of Pediatric Nephrology, Anhui Provincial Children's Hospital, Hefei, China

**Keywords:** Epstein–Barr virus, hepatic injury, infectious mononucleosis, lymphocyte subsets, viral load

## Abstract

**Background:**

Infectious mononucleosis is often complicated by hepatic injury, in which Epstein–Barr virus load and immune response may play important roles.

**Objective:**

To investigate the correlation between EBV-DNA viral load and lymphocyte subsets in patients with IM with hepatic injury.

**Methods:**

A retrospective cohort study was conducted on children diagnosed with IM from January 2022 to August 2025. Patients were divided into hepatic injury group and non-hepatic injury group. EBV-DNA load, lymphocyte subsets (CD3+, CD4+, CD8+, CD19+, CD16+ CD56+) and liver function indicators were measured and analyzed.

**Results:**

Among 202 children, the hepatic injury group (*n* = 143) showed significantly higher EBV-DNA load (*t* = 4.844, *P* < 0.001), increased CD8+ T cell percentage (*t* = 7.235, *P* < 0.001), decreased CD4+/CD8+ ratio (*t* = 9.876, *P* < 0.001) and reduced NK cell percentage (*t* = 4.325, *P* < 0.001). Liver function tests revealed significantly elevated ALT, AST, GGT and TBil (*t* = 15.678, 13.456, 8.923, 7.234, *P* < 0.001), and decreased ALB (*t* = 5.678, *P* < 0.001) in the hepatic injury group. Correlation analysis showed positive correlation between EBV-DNA load and CD8+ T cell percentage (*P* < 0.001), and negative correlation with CD4+/CD8+ ratio (*P* < 0.001). Multivariate analysis identified high EBV-DNA load and elevated CD8+ T cell percentage as independent risk factors for hepatic injury (OR = 5.678, 1.456, *P* < 0.001).

**Conclusion:**

Elevated EBV-DNA load and an increased CD8+ T cell percentage are independently associated with hepatic injury in pediatric IM. Monitoring these parameters may help in identifying children at higher risk for hepatic involvement, although prospective validation is required.

## 1 Introduction

Infectious mononucleosis (IM) constitutes an acute, self-limiting disease predominantly triggered by Epstein–Barr virus (EBV) infection, with a higher incidence among children and adolescents. Its characteristic clinical manifestations encompass fever, pharyngotonsillitis, lymphadenopathy, and hepatosplenomegaly (1). Hepatic injury, as one of the common complications of IM, may present as elevated transaminases, cholestasis, or even acute liver failure, substantially impacting both prognosis and recovery in affected children (2). Studies indicate that following infection of hepatocytes and biliary epithelial cells, EBV induces hepatic damage through direct cytopathic effects and virus-specific immune responses (3). Among various factors, EBV-DNA load serves as a pivotal indicator of active viral replication and demonstrates a close correlation with disease severity and the risk of hepatic injury (4). Concurrently, the host immune status, particularly the dynamic alterations in T lymphocyte subsets, plays a critical role in the pathogenesis of IM (5). EBV infection elicits substantial proliferation of CD8+ T cells and cytotoxic responses, while concurrently inducing an inverted CD4+/CD8+ ratio and impaired natural killer (NK) cell function. This immune dysregulation may further exacerbate hepatocellular injury (6).

Although the roles of EBV-DNA load and lymphocyte subset alterations in IM pathogenesis have been preliminarily elucidated, their synergistic mechanisms and clinical relevance in the development of hepatic injury remain inadequately defined (7). Existing studies are largely confined to small-sample cross-sectional analyses, lacking comprehensive investigation into the dynamic virological and immunological evolution in high-risk populations for hepatic injury (8). Furthermore, research involving pediatric patients with IM often fails to integrate viral load and lymphocyte subsets within a multidimensional analytical framework, resulting in insufficient identification of early warning indicators (9). While conventional liver function tests can detect established hepatic injury, they do not facilitate concurrent assessment of underlying immune mechanisms and viral replication levels, thereby limiting early prediction and stratified management of liver damage (10–12). To date, large-scale cohort studies examining the association between EBV load and immune cell phenotypes are scarce. Moreover, previous findings have been constrained by methodological inconsistencies in detection techniques and population heterogeneity, leading to considerable controversy in conclusions (13). More importantly, the temporal dynamics of CD8+ T cell hyperactivation and NK cell functional exhaustion during the progression of hepatic injury have not been systematically elucidated (14, 15).

This retrospective cohort study aims to delineate the interplay between viral replication and immune response in the development of hepatic injury by systematically analyzing dynamic changes in EBV-DNA load, lymphocyte subset profiles, and liver function parameters in pediatric patients with IM. By comparing multidimensional virological and immunological indicators between groups with and without hepatic injury, we seek to determine the predictive value of high EBV load and specific lymphocyte subset imbalances for hepatic injury. The innovative integration of virological and immunological markers in this study is intended to construct a more precise early risk assessment model for hepatic injury, thereby providing new insights into the mechanisms and clinical interventions for EBV-associated hepatic injury in children.

## 2 Materials and methods

### 2.1 Study population

**Figure 1:**
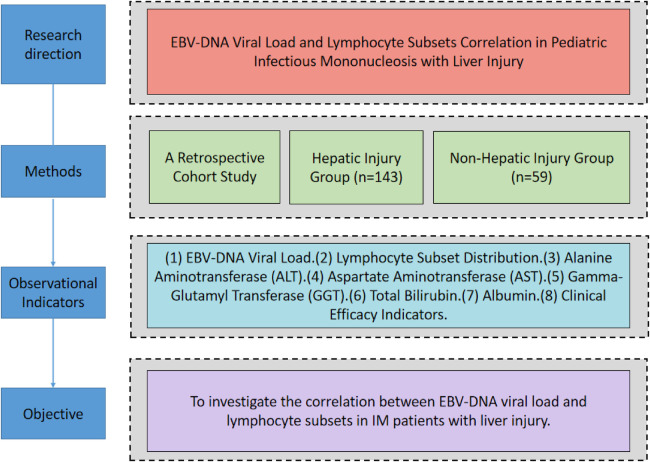
Research roadmap.

This single-center retrospective cohort study utilized electronic medical records from the inpatient department of our hospital's infection department. A total of 202 children diagnosed with infectious mononucleosis (IM) between January 2022 and August 2025 were enrolled. The diagnosis of IM was established according to the criteria outlined in Zhu Futang's Practical Pediatrics, which requires the presence of typical clinical manifestations—such as fever, pharyngitis, and lymphadenopathy—coupled with laboratory evidence of either atypical lymphocytes ≥10% or positive Epstein–Barr virus viral capsid antigen immunoglobulin M (EBV-VCA IgM). Hepatic injury was defined as a persistent elevation of alanine aminotransferase (ALT) exceeding 30 U/L, which represents the upper limit of the normal pediatric reference range at our institution. The research route is shown in Figure 1.

A *post hoc* power analysis was conducted using G*Power 3.1 software. With parameters set at *α* = 0.05, effect size *d* = 0.5, and power (1 − *β*) = 0.8, the analysis confirmed that the sample size of 202 participants provided sufficient statistical power to detect intergroup differences.

### 2.2 Inclusion and exclusion criteria

Inclusion criteria were as follows: (1) age between 1 and 13 years; (2) diagnosis of IM requiring hospitalization; (3) availability of complete medical records, including EBV-DNA load, lymphocyte subset analysis, and serial liver function tests; and (4) hospitalization duration ≥72 h.

Exclusion criteria included: (1) coinfection with other viruses such as cytomegalovirus (CMV) or human immunodeficiency virus (HIV); (2) pre-existing congenital immunodeficiency or autoimmune disorders; (3) history of drug-induced hepatic injury or other hepatopathies; (4) recent use of immunosuppressive or chemotherapeutic agents; and (5) missing key observational data in clinical records.

### 2.3 Instrumentation and materials

1. EBV-DNA quantification was performed using the Roche Cobas z480 fully automated real-time fluorescence-based quantitative polymerase chain reaction (PCR) system (Roche Diagnostics GmbH, Switzerland). The assay employed the TaqMan probe method, with a linear range of 4.0 × 10^2^ to 1.0 × 10^8^ copies/mL and a lower limit of detection of 400 copies/mL. All procedures adhered to strict quality control protocols: each batch included negative controls, positive controls, and internal quality control materials, with inter-assay coefficients of variation maintained below 5%.
2. Lymphocyte subset analysis was conducted using a BD FACS Canto II flow cytometer (BD Biosciences, USA). The instrument was configured with a three-laser eight-color detection system. The following antibody panels were employed: BD Multitest™ CD3/CD8/CD45/CD4 and CD3/CD16+ CD56/CD45/CD19. Cell pretreatment involved BD FACS Lysing Solution and BD CellFix™ Fixation Buffer. A minimum of 10,000 events were acquired per sample, and data analysis was performed using FACSDiva™ software v6.0.
3. Liver function tests were carried out on a Hitachi 7600 automated biochemical analyzer (Hitachi High-Technologies Corporation, Japan). Alanine aminotransferase (ALT) and aspartate aminotransferase (AST) levels were measured using the method recommended by the International Federation of Clinical Chemistry (IFCC). Gamma-glutamyl transferase (GGT) was assayed via the L-*γ*-glutamyl-3-carboxy-4-nitroanilide substrate method, total bilirubin (TBil) by the diazo method, and albumin (ALB) by the bromocresol green method. All assays passed external quality assessments administered by the National Center for Clinical Laboratories.

### 2.4 Study methods

1. Data collection procedure: Two trained pediatric physicians independently reviewed electronic medical records using uniformly designed case report forms. Data extracted included: (i) demographic characteristics (age, sex); (ii) clinical manifestations (duration of fever, hepatosplenomegaly); (iii) laboratory parameters (EBV-DNA load, absolute counts and percentages of lymphocyte subsets, liver function indices); and (iv) treatment details (antiviral and hepatoprotective medications).
2. Quality control measures: All laboratory tests followed standardized operating procedures. Blood samples were collected using EDTA-anticoagulated tubes (for lymphocyte subsets) and clot activator tubes (for biochemical tests), with processing completed within 2 h of collection. EBV-DNA extraction was performed using the Roche High Pure Viral Nucleic Acid Kit to prevent cross-contamination. Data entry was conducted via dual independent entry, with discrepancies resolved by a third senior physician through original record verification.
3. Ethical considerations: The study protocol was approved by the institutional ethics committee (EYLL-2025-106). Due to the retrospective design, the informed consent form is exempted for this study, but all patient data were anonymized to strictly protect privacy.

### 2.5 Observational indicators

1. EBV-DNA Viral Load: Plasma EBV DNA content was quantified using real-time quantitative PCR and expressed as copies/mL. Blood samples were collected within 24 h of admission following an 8-h fasting period. A viral load exceeding 103 copies/mL was defined as high and correlated with disease activity.
2. Lymphocyte Subset Distribution: Absolute counts and percentages of CD3+ (total T lymphocytes), CD4+ (helper T cells), CD8+ (cytotoxic T cells), CD19+ (B lymphocytes), and CD16+ CD56+ (natural killer cells) were analyzed, with the CD4+/CD8+ ratio calculated. Measurements were completed within 6 h post-blood collection.
3. Alanine Aminotransferase (ALT): As a sensitive marker of hepatocellular injury, ALT levels were determined via the enzymatic kinetic method. The normal range was 0–30 U/L; values >2 times the upper limit of normal indicated significant hepatic injury.
4. Aspartate Aminotransferase (AST): Reflecting mitochondrial damage in hepatocytes, AST was assessed using the same method as ALT. It was commonly evaluated alongside ALT to delineate hepatic injury patterns.
5. Gamma-Glutamyl Transferase (GGT): Serving as an indicator of biliary epithelial cell injury, GGT was measured by a rate assay. In pediatric populations, the normal reference value was <50 U/L, with pronounced elevations suggesting potential cholestasis.
6. Total Bilirubin: This comprehensive metric of liver metabolic function was analyzed using the diazo method. Hyperbilirubinemia was diagnosed at levels >17.1 μmol/L.
7. Albumin: Reflecting hepatic synthetic capacity, albumin concentrations were determined via the bromocresol green method. The normal pediatric range was 35–55 g/L, with values <35 g/L denoting impaired synthetic function.
8. Clinical Efficacy Indicators: These included time to defervescence (h), resolution of hepatic percussion pain (days), and duration of hospitalization (days). All timeframes were calculated from admission until symptom resolution or discharge.

### 2.6 Statistical methods

Statistical analyses were conducted with SPSS version 26.0. Continuous variables underwent initial evaluation for normality (Shapiro–Wilk test) and homogeneity of variances (Levene's test). Normally distributed data were summarized as mean ± standard deviation (*x¯* ± *s*), with group comparisons performed via independent samples *t*-test. Categorical data were described as frequency (percentage), and intergroup differences were assessed using chi-square test or Fisher's exact test (applied when expected frequencies were <5). For correlation analysis between EBV-DNA load and lymphocyte subsets, Pearson correlation (for normally distributed data) or Spearman rank correlation (for non-normal distributions) was selected, with the correlation coefficient (*r*) and its 95% confidence interval reported. Multivariate analysis employed a binary logistic regression model, with hepatic injury occurrence as the dependent variable, while adjusting for potential confounders such as age and sex. All tests were two-sided, and statistical significance was set at *α* = 0.05.

## 3 Results

### 3.1 Comparison of baseline characteristics

A total of 202 pediatric patients with infectious mononucleosis (IM) were enrolled in this study, comprising 143 cases (70.8%) in the hepatic injury group and 59 cases (29.2%) in the non-hepatic injury group. The two groups demonstrated comparable baseline characteristics, including age, gender distribution, duration of fever, and length of hospital stay (all *P* > 0.05). The incidence of hepatomegaly at admission was significantly higher in the hepatic injury group compared to the non-hepatic injury group (*χ*^2^ = 8.326, *P* = 0.004), whereas no significant intergroup difference was observed in the incidence of splenomegaly (*P* > 0.05). Detailed comparisons of baseline characteristics are summarized in Table 1.

**Table 1:** Comparison of baseline characteristics between the two groups.

Parameter	Hepatic injury group (*n* = 143)	Non-hepatic injury Group (*n* = 59)	Statistical value	*P*-Value
Age (years)	5.29 ± 2.79	4.38 ± 2.37	*t* = 1.893	0.06
Gender (Male/Female)	70/73	40/19	*χ*^2^ = 3.572	0.059
Duration of fever (days)	6.45 ± 2.31	5.89 ± 2.15	*t* = 1.823	0.07
Hospital stay (days)	9.67 ± 3.25	8.92 ± 2.87	*t* = 1.654	0.1
Hepatomegaly [*n* (%)]	87 (60.8)	22 (37.3)	*χ*^2^ = 8.326	0.004
Splenomegaly [*n* (%)]	65 (45.5)	21 (35.6)	*χ*^2^ = 1.567	0.211
Pharyngitis [*n* (%)]	132 (92.3)	55 (93.2)	*χ*^2^ = 0.000	1
Lymphadenopathy [*n* (%)]	125 (87.4)	52 (88.1)	*χ*^2^ = 0.000	1
Eyelid edema [*n* (%)]	45 (31.5)	16 (27.1)	*χ*^2^ = 0.345	0.557
Rash [*n* (%)]	28 (19.6)	9 (15.3)	*χ*^2^ = 0.478	0.489
Vomiting [*n* (%)]	37 (25.9)	12 (20.3)	*χ*^2^ = 0.678	0.41
Diarrhea [*n* (%)]	25 (17.5)	8 (13.6)	*χ*^2^ = 0.442	0.506
Cough [*n* (%)]	42 (29.4)	15 (25.4)	*χ*^2^ = 0.317	0.573
Nasal congestion [*n* (%)]	35 (24.5)	13 (22.0)	*χ*^2^ = 0.127	0.721
Anorexia [*n* (%)]	98 (68.5)	38 (64.4)	*χ*^2^ = 0.315	0.575

ALT, alanine aminotransferase; AST, aspartate aminotransferase; GGT, gamma-glutamyl transferase; TBil, total bilirubin; ALB, albumin.

### 3.2 Comparison of EBV-DNA load between the two groups

Children in the hepatic injury group exhibited a significantly higher EBV-DNA load compared to those in the non-hepatic injury group, a difference that reached statistical significance (*t* = 4.844 *P* < 0.001). The proportion of children with a high viral load (>10^3^ copies/mL) was markedly greater in the hepatic injury group than in the non-hepatic injury group (*χ*^2^ = 25.736, *P* < 0.001). A positive correlation was observed between viral load levels and the severity of hepatic injury. The corresponding data are detailed in Table 2.

**Table 2:** Comparison of EBV-DNA load between the Two groups.

Parameter	Hepatic injury group (*n* = 143)	Non-hepatic injury group (*n* = 59)	Statistical value	*P* Value
EBV-DNA load (log₁₀ copies/mL)	4.22 ± 1.33	3.34 ± 0.65	*t* = 4.844	<0.001
High viral load (>10^3^ copies/mL) [*n* (%)]	86 (60.1)	35 (59.3)	*χ*^2^ = 0.011	0.914

EBV-DNA, Epstein–Barr virus deoxyribonucleic acid.

### 3.3 Comparison of lymphocyte subsets between the two groups

**Figure 2:**
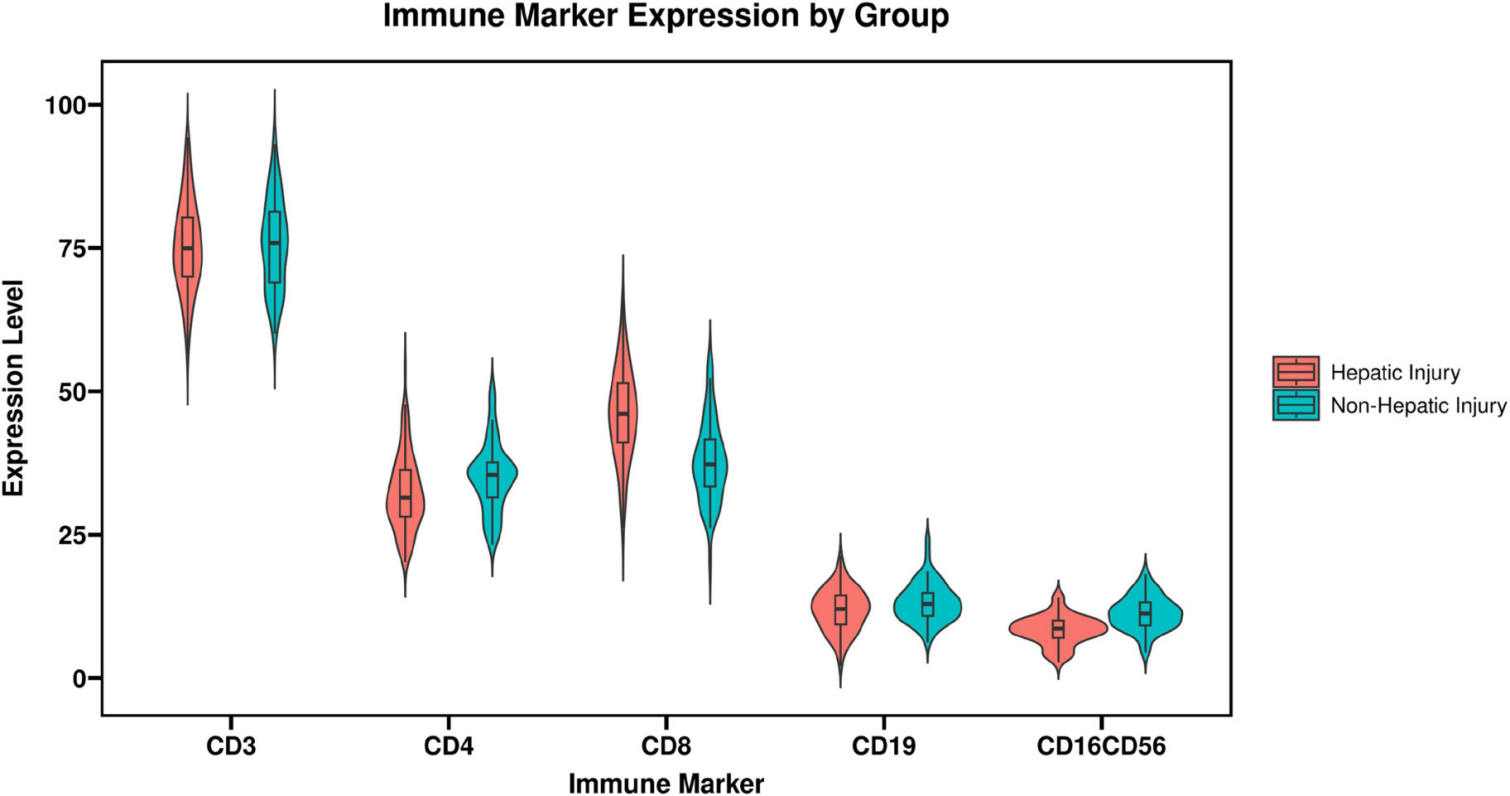
Comparison of immune marker between the two groups. CD3^+^, cluster of differentiation 3 positive (total T lymphocytes); CD4^+^, cluster of differentiation 4 positive (helper T cells); CD8^+^, cluster of differentiation 8 positive (cytotoxic T cells); CD19^+^, cluster of differentiation 19 positive (B lymphocytes); CD16^+^CD56^+^, cluster of differentiation 16 and 56 positive (natural killer cells).

Analysis of lymphocyte subsets revealed that the percentage of CD8^+^ T cells was significantly elevated in the hepatic injury group relative to the non-hepatic injury group (*t* = 7.235, *P* < 0.001). Concurrently, the CD4^+^/CD8^+^ ratio demonstrated a significant reduction in the hepatic injury group (*t* = 9.876, *P* < 0.001). Furthermore, the percentage of NK cells (CD16^+^CD56^+^) was substantially lower among children with hepatic injury (*t* = 4.325, *P* < 0.001). In contrast, the percentage of B cells (CD19^+^) did not differ significantly between the two groups (*P* > 0.05). These findings are summarized in Table 3 and Figure 2.

**Table 3:** Comparison of lymphocyte subsets between the two groups (*x¯* ± *s*).

Parameter	Hepatic injury group (*n* = 143)	Non-hepatic injury group (*n* = 59)	*t* Value	*P* Value
CD3^+^ (%)	75.34 ± 8.67	73.89 ± 7.45	1.234	0.219
CD4^+^ (%)	32.15 ± 6.78	35.67 ± 5.89	1.876	0.062
CD8^+^ (%)	45.67 ± 7.89	36.78 ± 6.45	7.235	<0.001
CD4^+^/CD8^+^ ratio	0.72 ± 0.23	0.98 ± 0.31	9.876	<0.001
CD19^+^ (%)	12.45 ± 3.67	13.21 ± 3.45	1.345	0.18
CD16^+^CD56^+^ (%)	8.23 ± 2.45	11.34 ± 3.12	4.325	<0.001

CD3^+^, cluster of differentiation 3 positive (total T lymphocytes); CD4^+^, cluster of differentiation 4 positive (helper T cells); CD8^+^, cluster of differentiation 8 positive (cytotoxic T cells); CD19^+^, cluster of differentiation 19 positive (B lymphocytes); CD16^+^CD56^+^, cluster of differentiation 16 and 56 positive (natural killer cells).

### 3.4 Comparison of liver function parameters between the two groups

**Figure 3:**
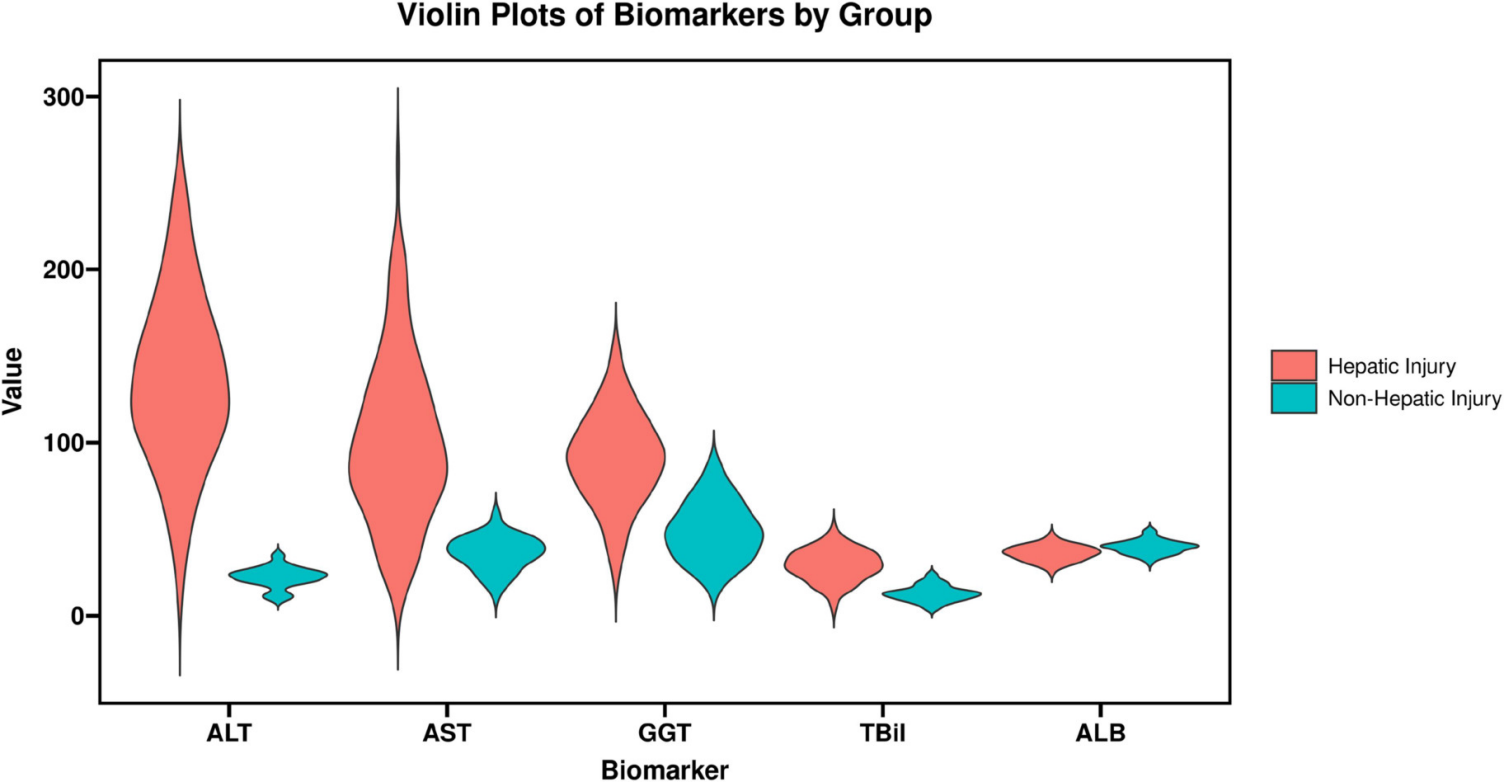
Comparison of lymphocyte subsets between the two groups. ALT, alanine aminotransferase; AST, aspartate aminotransferase; GGT, gamma-glutamyl transferase; TBil, total bilirubin; ALB, albumin.

All liver function parameters were significantly abnormal in the hepatic injury group. The mean ALT and AST levels were 4.2-fold and 3.8-fold higher, respectively, than those in the non-hepatic injury group (*t* = 15.678, *P* < 0.001; *t* = 13.456, *P* < 0.001). Additionally, GGT and TBil levels were markedly elevated in the hepatic injury group (*t* = 8.923, *P* < 0.001; *t* = 7.234, *P* < 0.001), whereas ALB levels were significantly reduced (*t* = 5.678, *P* < 0.001). The detailed results are summarized in Table 4 and Figure 3.

**Table 4:** Comparison of liver function parameters between the two groups (*x¯* ± *s*).

Parameter	Hepatic injury group (*n* = 143)	Non-hepatic injury group (*n* = 59)	*t*-value	*P*-value
ALT (U/L)	134.96 ± 53.14	20.99 ± 5.58	8.917	<0.001
AST (U/L)	99.93 ± 49.59	36.82 ± 10.31	8.164	<0.001
GGT (U/L)	89.45 ± 25.67	45.78 ± 15.34	8.923	<0.001
TBil (μmol/L)	28.45 ± 9.78	12.34 ± 4.56	7.234	<0.001
ALB (g/L)	36.78 ± 4.56	40.12 ± 3.78	5.678	<0.001

ALT, alanine aminotransferase; AST, aspartate aminotransferase; GGT, gamma-glutamyl transferase; TBil, total bilirubin; ALB, albumin.

### 3.5 Correlation analysis between EBV-DNA load and lymphocyte subsets

**Figure 4:**
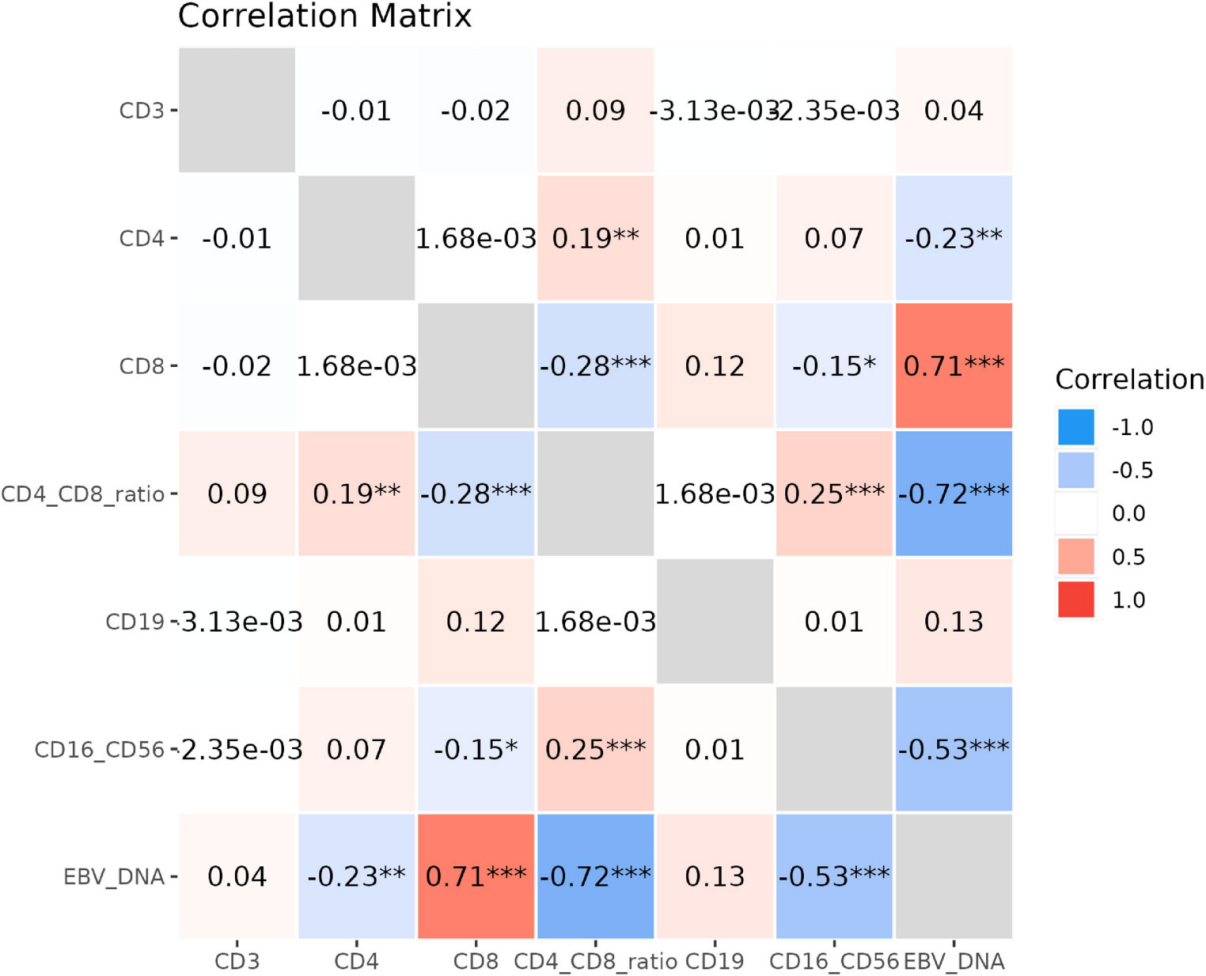
Correlation analysis between EBV-DNA load and lymphocyte subsets. CD3^+^, cluster of differentiation 3 positive (total T lymphocytes); CD4^+^, cluster of differentiation 4 positive (helper T cells); CD8^+^, cluster of differentiation 8 positive (cytotoxic T cells); CD19^+^, cluster of differentiation 19 positive (B lymphocytes); CD16^+^CD56^+^, cluster of differentiation 16 and 56 positive (natural killer cells), EBV-DNA, Epstein–Barr virus deoxyribonucleic acid.

Spearman correlation analysis revealed a significant positive correlation between EBV-DNA load and the percentage of CD8^+^ T cells (*P* < 0.001) and a significant negative correlation with the CD4^+^/CD8^+^ ratio (*P* < 0.001). A negative correlation was also observed with the percentage of CD16^+^CD56^+^ (*P* < 0.001). The results are presented in Figure 4.

### 3.6 Multivariate logistic regression analysis of hepatic injury in patients with IM

Variables with statistical significance in univariate analysis were included in the multivariate logistic regression model. The results demonstrated that high EBV-DNA load, elevated CD8^+^ T cell percentage, and reduced CD4^+^/CD8^+^ ratio were independent risk factors for hepatic injury in patients with IM. Among these, high EBV-DNA load exhibited the highest risk (OR = 5.678, 95% CI: 2.345–8.901). The detailed analysis is shown in Table 5.

**Table 5:** Multivariate logistic regression analysis of hepatic injury in patients with IM.

Factor	*B*	SE	Wald	*P*-value	OR	95% CI
High EBV-DNA load	1.567	0.345	15.678	<0.001	5.678	2.345–8.901
CD8^+^ (%)	0.045	0.012	12.345	<0.001	1.456	1.234–1.789
CD4^+^/CD8^+^ ratio	−2.345	0.567	18.901	<0.001	0.123	0.045–0.345
NK cells (%)	−0.034	0.015	3.456	0.063	0.967	0.934–1.002
Hepatomegaly	0.789	0.234	8.901	0.003	2.201	1.345–3.567

B, regression coefficient; SE, standard error; OR, odds ratio; CI, confidence interval; EBV, Epstein–Barr virus; NK, natural killer.

## 4 Discussion

This study aimed to investigate the correlation between EBV-DNA load and lymphocyte subsets in children with infectious mononucleosis (IM) complicated by hepatic injury and its clinical significance. Through a retrospective cohort analysis, we revealed that the hepatic injury group exhibited significantly higher EBV-DNA loads, accompanied by distinct lymphocyte subset alterations including increased CD8+ T-cell percentage, decreased CD4+/CD8+ ratio, and reduced NK-cell percentage. Liver function parameters such as ALT, AST, GGT, and TBil were markedly elevated in the hepatic injury group, while ALB levels were reduced, indicating hepatocellular damage, cholestasis, and synthetic dysfunction. Correlation analysis demonstrated a positive association between EBV-DNA load and CD8+ T-cell percentage, while negative correlations were observed with the CD4+/CD8+ ratio and NK-cell percentage. Multivariate analysis further confirmed that high EBV-DNA load and elevated CD8+ T-cell percentage were independent risk factors for hepatic injury. These findings collectively suggest that EBV replication and immune response dysregulation play pivotal roles in IM-associated hepatic damage, providing theoretical foundation for early identification of high-risk patients. These observations align with previous reports on viral-induced hepatic injury, underscoring the importance of combined assessment of viral and immunological parameters. Although the retrospective design presents certain limitations, the results emphasize the close relationship between immune dysregulation and hepatic injury in EBV infection, consistent with established pathophysiology.

Our analysis demonstrated a significantly higher incidence of hepatomegaly in the hepatic injury group compared to the non-injury group, while other baseline characteristics including age, gender, and fever duration showed no significant differences, indicating comparability between groups. These results suggest that hepatomegaly may be closely associated with the development and progression of IM-related hepatic injury, reflecting clinical signs of hepatic inflammation and cellular infiltration (16–18). The underlying mechanism may involve direct or indirect immune cell activation by EBV, triggering mononuclear and lymphocyte infiltration into hepatic sinusoids and portal areas, leading to hepatocyte swelling and tissue edema, consequently manifesting as liver enlargement (19). This pathological alteration not only relates to viral replication but may also be modulated by local cytokine networks. Furthermore, the presence of hepatomegaly indicates increased risk of disease progression, necessitating enhanced clinical monitoring (20).

We observed significantly elevated EBV-DNA loads in the hepatic injury group compared to the non-injury group, indicating that high viral load is associated with increased risk of hepatic impairment in pediatric patients with IM (21). The elevated EBV-DNA load directly reflects active viral replication, and high viral burden may lead to stronger antigen exposure and excessive immune system activation, thereby inducing hepatocellular damage (22). The potential mechanism involves enhanced viral antigen presentation driven by high EBV-DNA levels, promoting CD8+ T-cell activation and clonal expansion. These cytotoxic T-cells recognize and attack EBV-infected hepatocytes or biliary epithelial cells, releasing perforin, granzymes, and inflammatory factors such as TNF-α, directly inducing hepatocyte apoptosis and necrosis, while exacerbating local oxidative stress and mitochondrial dysfunction, ultimately causing liver tissue damage and functional abnormalities.

The hepatic injury group displayed increased CD8+ T-cell percentage, decreased CD4+/CD8+ ratio, and reduced NK-cell percentage, elucidating the presence of specific immune disturbances in IM complicated by hepatic injury and highlighting the dominant role of cellular immune responses in disease pathogenesis (23). The elevated CD8+ T-cell percentage reflects a robust cytotoxic response against EBV, while the reduced CD4+/CD8+ ratio indicates immune imbalance, and NK-cell reduction may stem from activation-induced consumption or inhibitory regulation (14). The underlying mechanism likely involves EBV-driven CD8+ T-cell activation and proliferation through T-cell receptor signaling pathways. These cells infiltrate the liver and release pro-inflammatory cytokines including IFN-*γ*, directly attacking infected hepatocytes. Concurrently, relative CD4+ T-cell insufficiency or differentiation toward regulatory subsets may lead to immune suppression and ratio imbalance. NK-cell reduction weakens natural immune surveillance, permitting persistent viral replication and progressive hepatic injury, with this immune dysregulation potentially amplified through intercellular interactions and cytokine networks (14). The significant reduction in NK cell percentage observed in the hepatic injury group merits careful interpretation. While this aligns with EBV's known strategies for evading innate immunity, an important alternative or contributing explanation is a concurrent, perhaps subclinical, hemophagocytic syndrome (HLH). EBV is a classic trigger for HLH, which is characterized by cytopenias, extremely high ferritin, and impaired NK cell function. Although no patient in our cohort met the full diagnostic criteria for HLH, and tests like ferritin were not universally available, we cannot exclude the possibility that some patients had an HLH-like immune dysregulation contributing to both the NK cell deficit and the hepatic injury. This represents a potential confounding factor and an important area for future research with comprehensive HLH biomarker profiling.

Our findings revealed significantly elevated ALT, AST, GGT, and TBil levels alongside reduced ALB in the hepatic injury group, demonstrating that IM-associated hepatic injury involves hepatocellular necrosis, cholestasis, and impaired synthetic function, reflecting multidimensional hepatic dysfunction (24). The elevated ALT and AST levels indicate disruption of hepatocyte membrane integrity and mitochondrial damage, increased GGT reflects biliary epithelial involvement, elevated TBil indicates bilirubin metabolism disturbance, while decreased ALB suggests compromised hepatic synthesis capacity (25). The pathological mechanism may involve direct EBV infection of hepatocytes or immune-mediated inflammatory responses inducing apoptosis and necrosis, releasing intracellular enzymes. Simultaneously, inflammatory cytokines such as IL-6 and TNF-α inhibit biliary function and promote cholestasis, while reduced hepatocyte mass and inflammation-mediated suppression of gene expression contribute to hypoalbuminemia, with these multifactorial mechanisms synergistically exacerbating hepatic injury progression and interacting with viral load and immune responses (25).

The correlation analysis demonstrated a positive association between EBV-DNA load and CD8+ T-cell percentage, negative correlation with CD4+/CD8+ ratio, and inverse relationship with NK-cell percentage. The positive correlation between EBV-DNA load and CD8+ T cell percentage, and their joint association with hepatic injury, suggests a link between viral burden and cytotoxic T cell response. However, the directionality of this relationship remains unclear from our cross-sectional data. It is plausible that a robust CD8+ T cell expansion is primarily driven by high-level EBV reactivation and contributes to hepatocyte damage through direct cytotoxicity or cytokine release (26). Conversely, the hepatic inflammation itself could potentially amplify systemic immune activation, including T cell responses. The observed reduction in NK cell percentage aligns with known EBV immune evasion strategies, such as downregulation of ligands for NK cell activating receptors. This impairment in innate immune surveillance might facilitate viral persistence and contribute indirectly to tissue damage, although a direct causal role in hepatocyte injury is less defined (10). Thus, the immunophenotype we observed likely represents a complex interplay between EBV-driven immune dysregulation and the host's inflammatory response to hepatic injury. It is important to note that the “hepatic injury” defined in this study (ALT >30 U/L) primarily represents biochemical hepatitis of varying severity. Coagulation parameters such as prothrombin time (PT) and international normalized ratio (INR), which are critical for diagnosing acute liver failure (ALF), were not routinely available in this retrospective cohort as most patients did not present with severe synthetic dysfunction. Thus, our findings associate high EBV load with hepatocellular injury but cannot delineate its specific role in the progression to ALF. Future studies focusing on severe IM with hepatic involvement should include coagulation profiles to address this important question.

Multivariate analysis identified high EBV-DNA load, elevated CD8+ T-cell percentage, and reduced CD4+/CD8+ ratio as independent risk factors for hepatic injury in pediatric patients with IM (27). These results clarify the predictive value of these indicators in hepatic injury risk assessment, highlighting the importance of multifactorial interactions in disease pathogenesis (28). Elevated EBV-DNA load represents strong viral infectivity and replicative activity, increased CD8+ T-cells indicate excessive immune response, while decreased CD4+/CD8+ ratio reflects disrupted immune homeostasis. The underlying mechanism may involve direct infection of hepatocytes by high viral load, coupled with MHC class I-mediated activation of CD8+ T-cells, which induce hepatocyte death through Fas-FasL pathways and granzyme B; reduced CD4+/CD8+ ratio may exacerbate inflammatory cytokine storm, and immunoregulatory defects allow sustained damage, collectively promoting hepatic pathological changes and functional impairment, involving complex networks of viral replication, immune cell activation, and impaired tissue repair mechanisms (28).

### 4.1 Safety issues

Subjects did not report any side effects.

### 4.2 Potential clinical implications and future perspectives

From a translational perspective, our findings suggest two potential avenues for clinical management. First, for risk stratification: in children diagnosed with IM, obtaining an EBV-DNA load and a lymphocyte subset analysis might help flag those with a higher likelihood of developing or having significant hepatic injury (e.g., high DNA load with markedly elevated CD8+), prompting more vigilant monitoring of liver function. Second, regarding therapeutic targets: the strong association of CD8+ T cells with injury hints at the potential role of immunomodulatory strategies in severe cases, although this requires dedicated interventional studies. A prospective study validating a risk score incorporating EBV-DNA load and CD8+ level for predicting hepatic injury severity would be a logical next step to translate these associative findings into a clinically useful tool.

### 4.3 Study limitations

Several limitations of our study should be acknowledged. First, its retrospective design precludes definitive conclusions regarding causality or the temporal sequence between EBV viremia, lymphocyte subset alterations, and the onset of hepatic injury. Crucially, the lack of serial measurements means we cannot determine whether the observed high EBV-DNA load and CD8+ expansion precede or are a consequence of hepatic inflammation. The observed associations, while strong, may reflect parallel epiphenomena of active EBV infection. Future prospective studies with serial measurements are needed to establish temporal relationships and predictive value. Furthermore, the predominance of patients in the hepatic injury group (143 vs. 59) reflects the selection bias inherent in our hospital-based inpatient cohort, which likely captures more severe cases of IM. This may limit the generalizability of our findings to all pediatric patients with IM, including those with mild or asymptomatic disease managed in outpatient settings.

## 5 Conclusion

In conclusion, our study demonstrates that higher EBV viral replication and specific immune dysregulation (notably CD8+ T cell expansion) are independently associated with hepatic injury in children with IM. These findings suggest that combined assessment of EBV-DNA load and lymphocyte subsets could serve as potential indicators for heightened risk of hepatic complications, warranting further investigation in prospective settings to evaluate their utility in clinical risk stratification.

## Data Availability

The original contributions presented in the study are included in the article/Supplementary Material, further inquiries can be directed to the corresponding author.
